# The Interactive Relationship between Pain, Psychosis, and Agitation in People with Dementia: Results from a Cluster-Randomised Clinical Trial

**DOI:** 10.1155/2016/7036415

**Published:** 2016-05-09

**Authors:** Torstein F. Habiger, Elisabeth Flo, Wilco P. Achterberg, Bettina S. Husebo

**Affiliations:** ^1^Department of Global Public Health and Primary Care, Centre for Elderly and Nursing Home Medicine, University of Bergen, 5018 Bergen, Norway; ^2^Department of Public Health and Primary Care, Leiden University Medical Center, 2300 RC Leiden, Netherlands; ^3^Municipality of Bergen, 5020 Bergen, Norway

## Abstract

*Background*. Neuropsychiatric symptoms are common in people with dementia, and pain is thought to be an important underlying factor. Pain has previously been associated with agitation, and pain treatment has been shown to ameliorate agitated behaviour. So far, the association between pain and psychosis and the effect of pain treatment on psychotic symptoms is unclear. Furthermore, the impact of opioid treatment on psychosis is not established.* Aim*. To investigate the efficacy of a stepwise protocol for treating pain (SPTP) on psychosis and agitation measured with the Neuropsychiatric Inventory, Nursing Home version, and to explore the impact of opioid analgesics on psychosis.* Method*. Secondary analyses are from a cluster-randomised controlled trial including 352 patients with advanced dementia and agitation from 18 nursing homes in Western Norway. The intervention group received pain treatment according to SPTP.* Results*. Pain was associated with disinhibition (adjusted OR: 1.21, 95% CI: 1.10–1.34) and irritability (adjusted OR: 1.10, 95% CI: 1.01–1.21) at baseline. Pain treatment reduced agitation (*p* < 0.001, df = 1; 300) and aberrant motor behaviour (*p* = 0.017, df = 1; 300). Psychosis was reduced in people with at least one symptom at baseline (*p* = 0.034, df = 1; 135). The use of opioid analgesics did not increase psychotic symptoms.* Study Registration*. This trial is registered with ClinicalTrials.gov (NCT01021696), Norwegian Medicines Agency, EudraCT (EudraCTnr: 2008-007490-20).

## 1. Introduction

Neuropsychiatric symptoms (NPS) are a feature in many neurodegenerative diseases, among other dementia, where over 90% of patients suffer from at least one NPS during the course of their disease [1]. NPS can be distressing for both patients and family alike and is often the main reason for admission to a nursing home (NH) [2]. NPS can be clustered in different ways. These clusters are most commonly defined by symptoms that present concurrently, like mood symptoms such as depression and anxiety, agitation symptoms such as aggression and irritability, and psychosis symptoms such as delusion and hallucination [3–6].

The aetiology of NPS is largely unknown, but factors like neuropathological changes in the brain, unmet psychosocial needs, and pain are thought to play a role [7]. Despite the multiple potential underlying factors, NPS are often treated with antipsychotic drugs with potential harmful side effects [8]. This highlights the importance of investigating the relationship between NPS and possible underlying treatable causes, such as pain, to avoid unnecessary antipsychotic drug use [9–11].

People in the later stages of dementia often reside in NHs and frequently experience pain, with 30–60% suffering daily from pain [12–14]. The cognitive decline with a subsequent loss of communicative abilities puts people with dementia at an increased risk of suffering from untreated pain [15, 16]. Research demonstrates that pain in people with dementia can act as a trigger for NPS such as agitation and mood symptoms [17, 18]. However, the relationship between pain and psychosis symptoms is less well studied, and only an association between pain and delusion has previously been described. Tosato et al. investigated the association between pain and NPS in NH patients with cognitive impairment and found pain to be associated with delusion [19]. In contrast, Cohen-Mansfield et al. found no association between pain and psychosis symptoms in an adult day care population (≥60 years old) residing in the community [20].

Our own research demonstrated the efficacy of individual pain treatment on behavioural disturbances in NH patients with advanced dementia and found that pain treatment ameliorated agitation as assessed by the Cohen-Mansfield Agitation Inventory (CMAI) [9]. Secondary analyses showed that pain treatment also reduced verbal aggression and restlessness [10]. Mood symptoms such as depression, sleep and appetite disturbances, measured with the Neuropsychiatric Inventory, Nursing Home version (NPI-NH) [11], and pain intensity assessed by the Mobilisation Observation Behaviour Intensity Dementia-2 (MOBID-2) Pain Scale [13] were also found to be reduced. The effect of pain treatment on psychosis and agitation symptoms measured by NPI-NH has, however, not yet been investigated.

Although there are no official guidelines for pain treatment in people with dementia, the use of opioid analgesics in pain treatment is recommended in guidelines for older people [21–23]. However, some physicians can be reluctant to prescribe these drugs, often due to the fear of possible side effects such as delirium, which also includes psychotic symptoms such as hallucination and delusion [24, 25]. The association between opioid analgesics and psychosis can therefore give relevant information regarding delirium as a potential side effect of opioid drug use.

The primary aim of this study was to investigate the efficacy of pain treatment on psychosis and agitation and the association between pain, psychosis, and agitation in people with advanced dementia. In addition, we investigated whether the use of opioid analgesics increased the prevalence of delusion and hallucination in people with dementia. We hypothesized an association between pain and agitation at baseline, but not between pain and psychosis, and suggested that pain treatment will reduce symptoms of agitation, but not symptoms of psychosis. We also hypothesized that the use of opioid analgesics does not increase the prevalence of hallucination and delusion.

## 2. Method

We conducted secondary analyses from a cluster-randomised controlled trial (RCT), investigating the efficacy of treating pain on behavioural disturbances in NH patients with advanced dementia from 18 NHs in Western Norway. For a more detailed description of the study procedure, we refer to previous publications [9, 11, 13]. In brief, patients included in this study had moderate to severe dementia as defined by the Diagnostic and Statistical Manual of mental disorders, 4th edition (DSM-IV); Functional Assessment Staging Test (FAST) score ≥ 4 [26]; Minimental State Examination (MMSE) score ≤ 20 [27], and clinically relevant behavioural disturbances as defined by a score ≥ 39 on CMAI [28]. Patients were excluded if they had an advanced medical disorder with expected survival ≤ 6 months, severe psychiatric or neurological disorder, hepatic or renal failure, a score ≥ 8 on the aggression item of the NPI-NH, with aggression as the predominant symptom [29], or allergy to paracetamol, morphine, buprenorphine, or pregabalin.

### 2.1. Study Design

Each NH unit was defined as a single cluster and was randomised to either intervention or control. Randomisation was performed by a statistician using Stata version 8, by generating a list of random numbers used for allocating each cluster to either intervention or control. The intervention group received individual pain treatment according to a stepwise protocol for treating pain (SPTP) for 8 weeks, followed by a 4-week washout period where analgesics were reverted back to preintervention treatment. The control group received treatment as usual. The SPTP was based on recommendations made by the American Geriatrics Society [22]. According to assessment of current medication and degree of pain, the patient was allocated to one of four steps, receiving either paracetamol (Paracetamol®), extended release morphine (Dolcontin®), buprenorphine transdermal patch (Norspan®) for patients with swallowing difficulties, or pregabalin (Lyrica®) for patients with suggested neuropathic pain. Physicians were instructed to keep the prescription unchanged if possible. Use of as-needed analgesics was not prohibited and was monitored during the study.

### 2.2. Outcome Measures

The primary outcome measure was NPS as measured by the NPI-NH [29]. The NPI-NH rates the frequency (*F*) and severity (*S*) of twelve different NPS. Frequency is rated on a scale from 1 to 4, where 1 represents occasionally (less than once a week) and 4 represents very frequent (daily or more often). Severity is measured on a scale from 1 to 3, where 1 represents mild (causes little stress for the patient) and 3 represents severe (puts very much stress on the patient and cannot easily be diverted by caregivers). The frequency and severity scores are multiplied (*F* × *S*) to give an item score for each NPS, where a score ≥ 4 was viewed as a clinically significant symptom [30].

The NPS measured by NPI-NH were clustered in three groups: agitation (aggression, disinhibition, irritability, and aberrant motor behaviour), psychosis (delusion, hallucination, and euphoria), and mood (depression, anxiety, apathy, and sleep and appetite disturbances), according to factor analyses by Cheng et al. [6].

Pain intensity was assessed by the MOBID-2 Pain Scale [31–33]. This is a nursing staff-administered pain tool, consisting of two parts. The first part assesses pain originating from the musculoskeletal system during five active guided movements. The second part assesses pain that might be related to internal organs, head, and skin based on the caregivers' observation during the last week. Taking all items into account, the caregiver rated the patients' pain on a Numerical Rating Scale (NRS) ranging from 0 to 10, where 0 represented no pain and 10 the worst pain imaginable. This tool has been thoroughly tested for its psychometric properties and showed good validity, reliability, and responsiveness [32, 33].

All assessments were conducted at baseline and Weeks 2, 4, 8, and 12 by the primary caregivers who knew the patient best in collaboration with a specialised study nurse.

### 2.3. Statistics

Differences in baseline characteristics were explored using an independent sample* t*-test for normally distributed variables; a Chi-squared test was used for categorical variables, and a Mann-Whitney* U* test was used for nonparametric variables. Associations between pain, psychosis, and agitation at baseline were investigated by using crude and adjusted logistic regression. Each symptom of psychosis and agitation represented the dependent variable, while total pain intensity, assessed by MOBID-2, represented the explanatory variable. Associations were adjusted for age, gender, dementia severity (assessed by MMSE and FAST), and activities of daily living (ADL) function assessed by Barthels ADL index [34]. The changes in *F* × *S* score between the intervention and control groups from baseline to Week 8 were compared using the Mann-Whitney* U* test. The association between opioid analgesics and delusion and hallucination was evaluated at baseline and Week 8 using logistic regression. Associations were adjusted for age, gender, dementia severity (MMSE and FAST), ADL function (Barthels ADL index), and pain intensity (MOBID-2). Statistic calculations were performed using the Statistical Package for Social Sciences (SPSS) version 22.

## 3. Ethics

Informed consent was obtained from patients who were cognitively able to understand the possible risks and benefits of the study. Consent was, if possible, obtained in a meeting where next of kin was present as well. A presumed consent was obtained from next of kin, or a legal guardian, if the patient was not able to give an informed consent. All consents were obtained in accordance with local law, approved by the Regional Ethical Committee for Medical Ethics in Western Norway (REK-Vest 248.08), and authorised by the participating institutions' review board.

## 4. Results

Three hundred and fifty-two patients from 60 NH units were included. Units were randomised to either intervention or control, generating 177 patients in the control group and 175 patients in the intervention group. With the exception of age (*p* = 0.022), we found no differences between the two groups. Baseline characteristics are described in Table 1. During the intervention period, 13 patients in the control and 25 in the intervention group were excluded, with no significant differences between the two groups [9]. At baseline, 71 people in the control group (40%) and 83 people in the intervention group (47%) had one or more symptoms of psychosis, while 128 people in the control group (72%) and 137 people in the intervention group (78%) had one or more symptoms of agitation. The most prevalent symptom was irritability (48%), while the least prevalent one was euphoria (9%).

**Figure 1:**
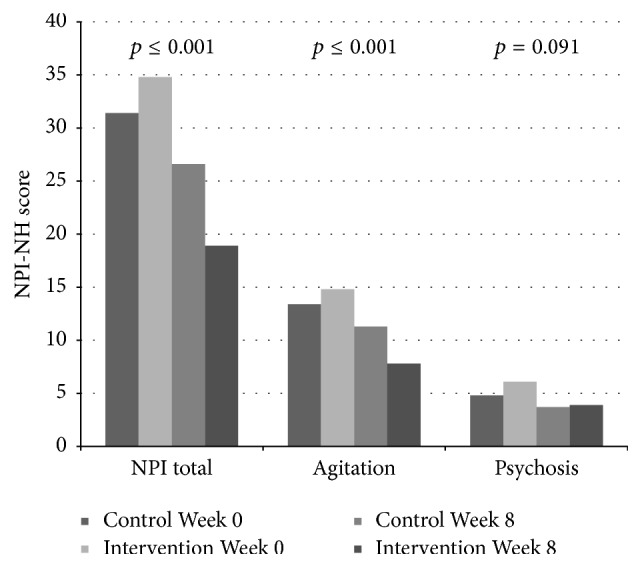
The efficacy of treating pain on psychosis and agitation.

**Figure 2 fig2:**
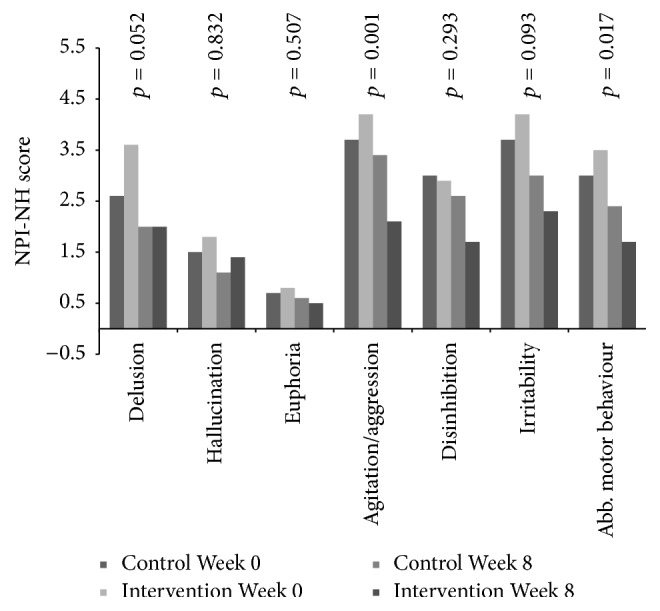
The efficacy of pain treatment on individual neuropsychiatric symptoms.

**Figure 3:**
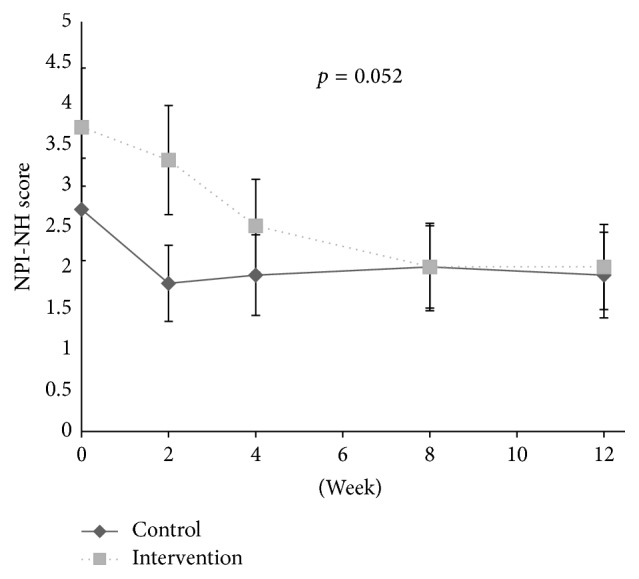
Development of delusion during the intervention and washout period.

Related to symptoms of psychosis, no associations were found between pain and symptoms of psychosis at baseline. During the intervention period, no reduction in the psychosis cluster (*p* = 0.091, df = 1; 300), delusion (*p* = 0.052, df = 1; 300), hallucination (*p* = 0.832, df = 1; 300), and euphoria (*p* = 0.507, 1; 300) was observed in response to individual pain treatment compared to the control group from baseline and to Week 8 (Table 2, Figures 1 [Fig fig2]–3). However, for people with one or more symptoms of psychosis at baseline, a decrease was observed in the psychosis cluster (*p* = 0.034, df = 1; 135) and delusion (*p* = 0.031, df = 1; 135) in the intervention group compared with the control group (Table 3, Figure 7).

**Figure 4:**
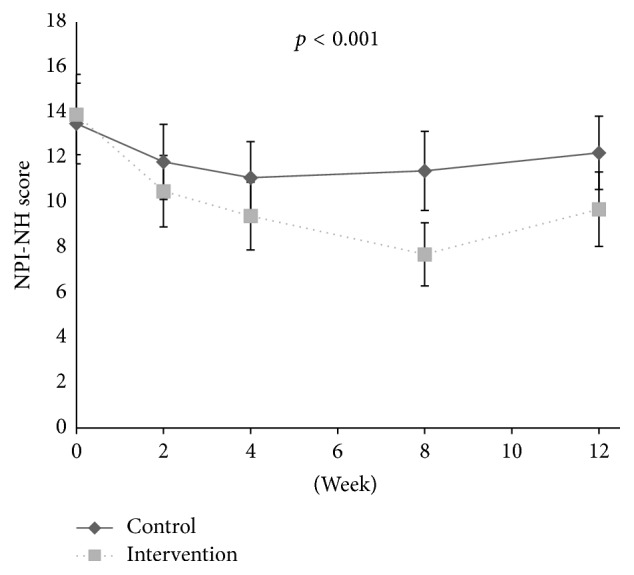
Development of agitation scores in clusters during intervention and washout period.

**Figure 5:**
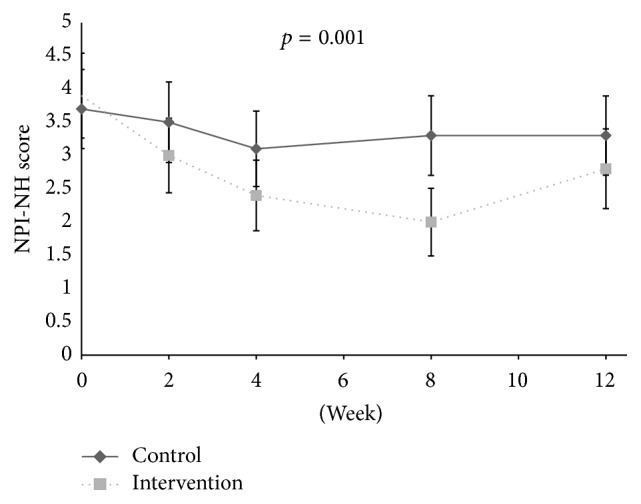
Development of agitation/aggression during the intervention and washout period.

**Figure 6:**
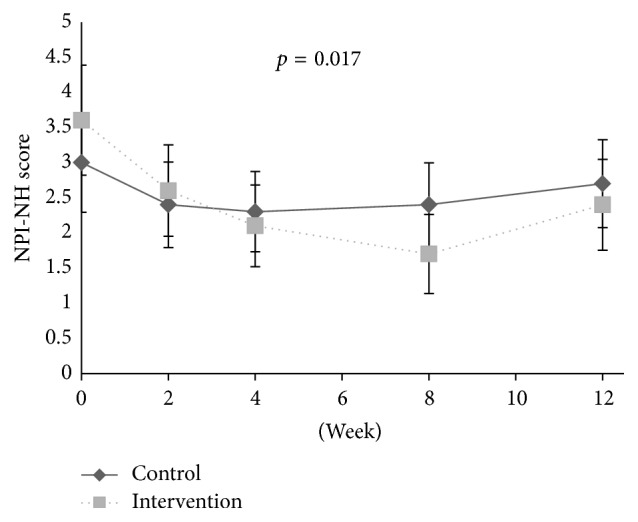
Development of aberrant motor behaviour during the intervention and washout period.

**Figure 7:**
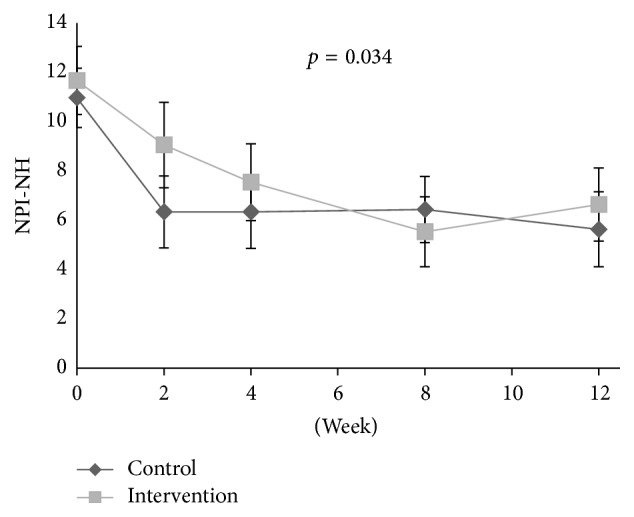
Development of the psychosis cluster in patients with one or more clinically significant NPS of psychosis at baseline (NPI-NH ≥ 4).

**Figure 8:**
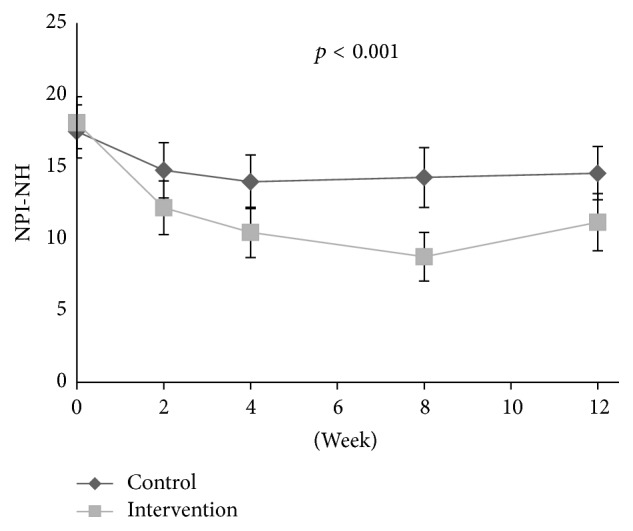
Development of the agitation cluster in patients with one or more clinically significant NPS of agitation at baseline (NPI-NH ≥ 4).

At baseline, the adjusted logistic regression analysis showed a positive association between disinhibition and level of pain (OR: 1.18, aOR: 1.21, 95% CI: 1.10–1.34, and* p* < 0.001) and between irritability and level of pain (OR: 1.11, aOR: 1.10, 95% CI: 1.01–1.21, and* p* = 0.032), adjusted for confounders. During the intervention period, a decrease in the agitation cluster (*p* < 0.001, df = 1; 301), agitation/aggression (*p* = 0.001, df = 1; 301), and aberrant motor behaviour (*p* = 0.017, df = 1; 301) was found in the treatment group compared to the control group (Table 2, Figures 1, 2, 4, 5, and 6). For people with one or more symptoms of agitation at baseline, a decrease during the intervention period was observed in the agitation cluster (*p* < 0.001, df = 1; 228), agitation/aggression (*p* = 0.004, df = 1; 228), and aberrant motor behaviour (*p* = 0.007, df = 1; 228) in the treatment group compared with the control group (Table 3, Figure 8).

At baseline, the use of opioid analgesics was not associated with the prevalence of delusions (OR: 0.97, aOR: 0.96, 95% CI: 0.56–1.65, and* p* = 0.870) or hallucination (OR: 0.76, aOR: 0.69, 95% CI: 0.34–1.41, and* p* = 0.314). Following the intervention period at Week 8, opioids were not associated with the prevalence of delusion (OR: 1.90, aOR: 1.89, 95% CI: 0.72–4.98, and* p* = 0.200) or hallucination (OR: 1.05, aOR: 1.26, 95% CI: 0.39–4.09, and* p* = 0.700).

## 5. Discussion

This study aimed to investigate the relationship between pain, psychosis, and agitation, the efficacy of treating pain on psychosis and agitation, and the potential impact of opioid analgesics on the development of hallucination and delusion in NH patients with advanced dementia.

The study showed that treatment of pain ameliorates the prevalence of psychosis and delusion in people with dementia who presented at least one psychosis symptom at baseline. It is also established that, in this study, opioid analgesics did not increase the prevalence of hallucination or delusion. These findings confirmed the hypothesis that pain is a potential underlying cause for psychosis and that proper pain management is needed in order to avoid psychotic symptoms. This provides important information for clinicians when pharmacological treatment options for pain are to be evaluated. Some clinicians can be reluctant to prescribe opioid analgesics for pain treatment of people with dementia, often due to fear of anticholinergic side effects, such as delirium [24]. Finally, we found that pain treatment reduced agitation, aggression, and aberrant motor behaviour. This underlines previous findings where pain was found to be an important underlying cause for agitation assessed with CMAI in people with dementia. These findings highlight the fact that proper pain assessment should be a prerequisite when deciding treatment options for agitation in people with dementia.

The current study was the first parallel group-controlled trial investigating the efficacy of analgesics on psychotic symptoms in people with advanced dementia. Although individual pain treatment reduced psychosis in people with psychotic symptoms, pain was, interestingly, not cross-sectionally associated with hallucination and delusion at baseline. Tosato et al. used data from the Minimum Data Set (MDS) and investigated the relationship between pain and psychiatric symptoms in 2822 NH residents with cognitive impairment and found an association between pain and delusion but not between pain and hallucination [19], contrary to our results. In Tosato's study, the interRAI MDS 2.0 instrument for long-term facilities was used to measure psychosis and pain, while our study used the MOBID-2 Pain Scale to measure pain. Cohen-Mansfield et al. also investigated the association between pain, delusion, and hallucination in an adult day care population and found no association between pain and delusion or pain and hallucination [20]. However, in contrast to our study, these people were not residing in NHs and patients suffering from dementia were not analyzed as a separate group. The study used the Behavioural Pathology in Alzheimer's disease rating scale to measure psychosis and a questionnaire, based on the short form of the McGill Pain Questionnaire, distributed to family and caregivers to measure pain. Pain should be measured by a tool thoroughly tested for psychometric properties, and among the measurement tools used, only MOBID-2 has been tested for validity, reliability, and responsiveness [32, 33].

We used a symptom clustering largely based on a factor analyses of the NPI-NH by Cheng et al., where the symptoms were clustered in three main groups: agitation, mood, and psychosis [6]. This clustering makes “clinical sense” and is in line with other previous studies. Hollingworth et al. grouped delusion and hallucination in a psychosis cluster, aggression and irritability in an agitation cluster, and disinhibition, euphoria, and aberrant motor behaviour in a behavioural dyscontrol cluster [3]. In a four-factor solution, Selbæk and Engedal grouped hallucination and delusion as a psychosis cluster and aggression, irritability, disinhibition, and aberrant motor behaviour in an agitation cluster [4]. Overall, the clusters may be viewed as merely theoretical constructs and changes assessed over time [4].

The reduction in psychosis was largely attributed to the reduction of delusion, as neither hallucination nor euphoria was reduced in response to pain treatment. This indicates that hallucination and euphoria may not be associated with pain. Traditionally, antipsychotics are recommended for short-time treatment of psychosis, also in people with dementia, despite potential harmful side effects and increased mortality [8]. Our results suggested that hallucination and euphoria were not associated with pain, making the use of antipsychotics in treatment of hallucination and euphoria more warranted than in treatment of delusion.

The use of opioid analgesics did not increase the prevalence of delusion or hallucination at baseline, or after the 8-week intervention. This is of key importance, because opioid analgesics such as morphine or buprenorphine can have multiple side effects such as confusion and delirium caused by anticholinergic activity [24]. Notably, delirium, psychosis, and depression have several similarities in people with dementia, making them difficult to distinguish and diagnose. This highlights the importance of trained staff in order to discriminate between the more acute state delirium and more chronic symptoms in dementia [25].

The reduction of agitation in response to pain treatment was fairly expected, as previous analyses on the study population have shown a decrease in behavioural disturbances, especially agitation, as measured using CMAI [9, 10]. NPI-NH does however measure more specific symptoms in contrast to CMAI, which measures more specific behavioural items. Therefore, the efficacy of pain treatment on the specific symptom aberrant motor behaviour is an interesting finding, supported by previous studies which found that pain treatment may reduce agitation. An article by Flo et al. reviewed studies on pain management in people with dementia and found that pharmacological pain treatment could reduce agitation [17]. Achterberg et al. reviewed the efficacy of pain management in people with dementia and found that pain can be a possible underlying cause for agitation and that a thorough pain assessment and management can ameliorate agitation [16]. The present analyses also found that there was an association between pain and disinhibition and irritability at baseline. While previous studies have found an association between pain and agitation, the direct association between pain, disinhibition, and irritability has not previously been described [17, 18, 35]. Our results showed that NPS associated with pain at baseline, like irritability and disinhibition, were not reduced in response to pain treatment. Results also showed that NPS not associated with pain at baseline, like agitation and delusion, were reduced in response to pain treatment. This paradox simply highlights the complex aetiology of NPS of agitation, and a thorough assessment of all possible underlying causes is important when deciding on possible treatment options for neuropsychiatric symptoms in people with dementia. Pain and behaviour are strongly intertwined, and the efficacy of both behavioural interventions and pain medication can improve both pain and behaviour [36].

*Strengths and Limitations*. This is the first RCT investigating the efficacy of treating pain on psychosis. Results came from secondary analyses from a previous study where CMAI was the primary outcome and NPI-NH was a secondary outcome. Inclusion criteria were therefore based on behavioural disturbances measured using CMAI. The number of study participants was also a limitation, as the group of patients with psychosis at baseline were a subgroup of the original population and a small sample. Despite this, the study is still the largest RCT investigating the efficacy of treating pain on psychosis and agitation.

## 6. Conclusion

Pain seems to be an underlying cause of psychosis and especially delusion. In addition, pain seems to be an underlying cause of agitation, such as aberrant motor behaviour. Thus, proper pain assessment is needed when treating these symptoms in people with dementia. The use of opioid analgesics does not seem to increase the prevalence of delusion and hallucination; therefore, the reluctance to use them may not necessarily be to the benefit of the patient.

## Figures and Tables

**Table 1:** Sample characteristics of patients at baseline.

	Control (*n* = 177)	Intervention (*n* = 175)	df	*p*
Age (SD)^a^	86.5 (6.7)	84.9 (7.0)	350	0.022
Women (%)^b^	131 (74.0)	131 (74.9)	1	0.856
FAST (SD)^c^	6.0 (0.7)	6.1 (0.7)	349	0.057
MMSE (SD)^c^	8.4 (6.7)	7.5 (6.5)	346	0.177
Barthels ADL total score (SD)^c^	8.6 (5.6)	7.9 (5.7)	339	0.216
CMAI total score (SD)^c^	56.2 (16.1)	56.5 (15.2)	349	0.487
MOBID-2 (SD)^c^	3.7 (2.5)	3.8 (2.7)	325	0.988
Medications (SD)^c^	3.6 (1.6)	3.4 (2.1)	318	0.146
Analgesics (%)^b^	122 (68.9)	117 (66.9)	1	0.404
Paracetamol (%)^b^	94 (53.1)	99 (56.6)	1	0.665
Opioids (%)^b^	51 (28.8)	43 (24.6)	1	0.292
NSAIDS (%)^b^	9 (5.1)	13 (7.4)	1	0.364
Psycholeptics (%)^b^	112 (63.3)	104 (59.4)	1	0.458
Antipsychotics (%)^b^	13 (7.3)	17 (9.7)	1	0.465
Anxiolytics (%)^b^	86 (48.6)	80 (45.7)	1	0.589
Psychosis symptoms (%)^b^	71 (20.2)	83 (23.6)	1	0.209
Delusion (%)^b^	49 (27.7)	66 (37.7)	1	0.056
Hallucination (%)^b^	29 (16.4)	32 (18.3)	1	0.690
Euphoria (%)^b^	15 (8.5)	16 (9.1)	1	0.864
Agitation symptoms (%)^b^	128 (36.4)	137 (38.9)	1	0.285
Agitation/aggression (%)^b^	74 (41.8)	85 (48.6)	1	0.253
Disinhibition (%)^b^	56 (31.6)	59 (33.7)	1	0.760
Irritability (%)^b^	84 (47.5)	85 (48.6)	1	0.956
Aberrant motor behaviour (%)^b^	57 (32.2)	65 (37.1)	1	0.388

^a^Independent-samples *t*-test.

^b^Pearson's Chi-squared test.

^c^Mann-Whitney *U* test.

**Table 2:** Efficacy of treating pain on psychosis and agitation.

	Baseline			8 weeks			
	Control (*n* = 177)	Intervention (*n* = 175)	*p* ^a^	Control (*n* = 157)	Intervention (*n* = 146)	*p* ^a^	*p* change^b^
NPI total score	31.4 (21.4)	34.8 (21.9)	0.132	26.6 (20.1)	18.9 (17.5)	<0.001	<0.001
Psychosis cluster	4.8 (5.8)	6.1 (6.9)	0.087	3.7 (4.9)	3.9 (5.5)	0.682	0.091
Delusion	2.6 (3.8)	3.6 (4.3)	0.030	2.0 (3.1)	2.0 (3.2)	0.813	0.052
Hallucination	1.5 (2.9)	1.8 (3.2)	0.427	1.1 (2.3)	1.4 (2.7)	0.405	0.832
Euphoria	0.7 (2.0)	0.8 (2.2)	0.887	0.6 (1.9)	0.5 (1.8)	0.123	0.507
Agitation cluster	13.4 (10.9)	14.8 (10.9)	0.155	11.3 (10.9)	7.8 (8.3)	0.007	<0.001
Agitation/aggression	3.7 (3.9)	4.2 (4.3)	0.373	3.4 (3.8)	2.1 (3.1)	0.001	0.001
Disinhibition	3.0 (4.0)	2.9 (3.8)	0.922	2.6 (3.9)	1.7 (3.0)	0.061	0.293
Irritability	3.7 (3.7)	4.2 (4.1)	0.338	3.0 (3.4)	2.3 (3.1)	0.092	0.093
Abb. motor behaviour	3.0 (4.5)	3.5 (4.7)	0.328	2.4 (3.7)	1.7 (3.6)	0.052	0.017

^a^Calculated by analyzing the difference between the intervention group and control group at each measurement point using the Mann-Whitney *U* test.

^b^Calculated by analyzing the difference in change of NPI-NH score in the intervention group versus the control group from baseline to Week 8 using the Mann-Whitney *U* test.

**Table 3:** Efficacy of treating pain on psychosis and agitation in patients presenting one or more clinically significant symptoms at baseline (NPI-NH ≥ 4).

	Baseline (SD)			8 weeks (SD)			
	Control (*n* = 71)	Intervention (*n* = 83)	*p* ^a^	Control (*n* = 67)	Intervention (*n* = 70)	*p* ^a^	*p* change^b^
Psychosis cluster	10.5 (4.7)	11.6 (5.9)	0.314	6.4 (5.3)	5.6 (6.1)	0.148	0.034
Delusion	5.6 (4.2)	6.9 (4.0)	0.043	3.2 (3.7)	2.9 (3.6)	0.770	0.031
Hallucination	3.2 (3.8)	3.3 (4.0)	0.813	2.1 (3.1)	2.1 (3.3)	0.987	0.925
Euphoria	1.7 (2.9)	1.4 (3.1)	0.211	1.0 (2.2)	0.5 (1.9)	0.027	0.758

	Control (*n* = 128)	Intervention (*n* = 137)	*p* ^a^	Control (*n* = 117)	Intervention (*n* = 113)	*p* ^a^	*p* change^b^
Agitation cluster	17.4 (9.7)	18.0 (9.6)	0.422	14.0 (11.0)	8.8 (8.8)	<0.001	<0.001
Agitation/aggression	4.7 (4.0)	5.1 (4.2)	0.441	4.2 (4.0)	2.5 (3.3)	0.001	0.004
Disinhibition	3.9 (4.3)	3.5 (4.0)	0.618	3.3 (4.2)	1.9 (3.2)	0.008	0.211
Irritability	4.8 (3.6)	5.1 (4.1)	0.664	3.6 (3.6)	2.6 (3.2)	0.023	0.183
Abb. motor behaviour	4.0 (4.7)	4.3 (4.9)	0.639	2.9 (3.9)	1.8 (3.5)	0.008	0.007

^a^Calculated by analyzing the difference between the intervention group and control group at each measurement point using the Mann-Whitney *U* test.

^b^Calculated by analyzing the difference in change of NPI-NH score in the intervention group versus the control group from baseline to Week 8 using the Mann-Whitney *U* test.
